# Kidney growth following preterm birth: evaluation with renal parenchyma ultrasonography

**DOI:** 10.1038/s41390-022-01970-8

**Published:** 2022-02-04

**Authors:** Sonja Brennan, David L. Watson, Donna M. Rudd, Yogavijayan Kandasamy

**Affiliations:** 1 Ultrasound Department, Townsville University Hospital, Townsville, QLD Australia; 2 Division of Tropical Health and Medicine, James Cook University, Townsville, QLD Australia; 3 Maternal Fetal Medicine Unit and Department of Obstetrics and Gynaecology, Townsville University Hospital, Townsville, QLD Australia; 4 Department of Neonatology, Townsville University Hospital, Townsville, QLD Australia; 5 Mothers and Babies Research Centre, Hunter Medical Research Institute, John Hunter Hospital, The University of Newcastle, Newcastle, NSW Australia

## Abstract

**Background:**

Preterm birth impairs nephrogenesis, leading to a reduced nephron endowment which is inextricably linked to hypertension and chronic kidney disease in adults. The aim of this study was to compare nephron endowment between preterm infants to that of intrauterine fetuses at the same gestational age (GA) using a novel indirect ultrasound measurement of the renal parenchymal thickness. We hypothesized that extrauterine and intrauterine renal parenchymal thickness would differ based on altered renal growth environments.

**Methods:**

In this observational study, appropriately grown preterm infants (birth weight of between the 5th and 95th percentile) born <32 weeks, admitted to the neonatal department were eligible to participate. Renal parenchymal thickness of the infants was measured at 32- and 37-weeks postmenstrual age (PMA). These measurements were compared to the intrauterine renal parenchymal thickness of appropriately grown fetuses (control).

**Results:**

At 32-weeks PMA, the preterm infants had a significantly thinner renal parenchyma compared to fetuses at 32-weeks GA suggesting they had less nephrons, however by 37-weeks there was no significant difference in renal parenchymal thickness.

**Conclusions:**

We propose that the differences in the extrauterine growth of the renal parenchyma in preterm infants may be due to a reduced number of nephrons and compensatory hyperfiltration.

**Impact:**

- This article provides insight into the effects of prematurity on nephrogenesis by comparing extrauterine renal parenchymal growth of born preterm infants to the ideal intrauterine fetal growth.
- Renal parenchyma thickness measurement using ultrasonography is a novel non-invasive measurement of renal development for the determination of nephron endowment.
- Differences in the renal parenchymal thickness of the preterm infants may be due to a deficit in nephron number and compensatory hyperfiltration.

## Introduction

Preterm birth is inextricably linked to impaired nephrogenesis and reduced nephron endowment in infancy, leading to hypertension and chronic kidney disease (CKD) in adults.^1–5^ With improving survival of preterm infants at earlier and earlier gestations we must also consider the impact of birth on the premature kidneys and long-term kidney function. Nephrogenesis continues in-utero until 36 weeks and therefore in preterm infants it is still in progress, to some degree, in the extrauterine environment.^6,7^ Studies have demonstrated that the extrauterine environment appears to adversely affect nephrogenesis, resulting in less layers of nephrons formed and an increased number of maldeveloped and dysfunctional glomeruli within the nephrons.^7,8^ Finally, during life, additional insults to the kidneys may further decrease nephron number making the development of CKD and hypertension much more likely, than for those born with a full complement of nephrons.^9,10^ Data from animal and human autopsy studies have demonstrated postnatal extrauterine nephrogenesis in preterm infants is different to normal intrauterine kidney development, with preterm infants having fewer functional nephrons.^7,11,12^ The challenge is to be able to assess nephron number non-invasively.

Ultrasound is the primary imaging modality for the assessment of neonatal kidneys as it is non-invasive, lacks ionizing radiation, mobile, widely available, and relatively inexpensive compared to other imaging.^13^ Renal volume, derived from measurement of the length, width, and height of the kidney, is used as a method to estimate nephron number,^5,14,15^ however, renal volume includes measurement of the parenchyma and the intrarenal collecting system. In the setting of hydronephrosis, a volume measurement would overestimate the true volume of kidney tissue and the kidney may actually have thinning of the parenchyma and a reduced nephron endowment. We have previously demonstrated that measuring only the renal parenchyma has less variance than renal volume and appears to be a more accurate method to estimate nephron number.^16,17^ There are currently, to our knowledge, no studies comparing renal parenchymal growth between preterm delivered infants and undelivered normally grown fetuses who would be expected to achieve optimal renal parenchymal growth and ideal nephron endowment.

The aim of this study was to compare the extrauterine growth of the renal parenchymal thickness in preterm infants to intrauterine renal parenchymal growth of appropriately grown fetuses at the same gestation. This study uses an ultrasound measurement of the renal parenchyma as an indirect estimate of in-vivo nephron number. As preterm birth is thought to impair nephrogenesis, we hypothesized that extrauterine renal parenchymal thickness is different from intrauterine fetal renal parenchymal thickness.

## Methods

### Study population

Townsville University Hospital’s tertiary perinatal center provides care to the North Queensland region and has a catchment population of around 700,000.^18^ This prospective, observational study was conducted in the Ultrasound and Neonatal Departments between August 2010 and October 2018. The study was approved by the Townsville Hospital and Health Service Human Research Ethics Committee. In this manuscript, we are comparing the data from preterm delivered infants (study) and fetal (control) cohorts at an equivalent GA. We utilized data from two different cohorts (preterm and fetal data of term infants) obtained from parallel studies carried out by the research team. Preterm infants recruited for this study were a part of a larger study that evaluated the association between prematurity, retinal vascularization, and renal development in preterm and low birth weight infants.^15,19^ The fetal cohort was from a parallel study that investigated fetal renal growth.^20^

Preterm infants born at less than 32-weeks of gestation, who were admitted to the neonatal department during the study period were classified as the study population. For this study, just the appropriately grown preterm infants (AG) (birth weight was between the 5th and 95th percentile on a fetal weight chart^21^) were included. A fetal weight was used as it represents the normal intrauterine growth velocity.^22,23^ The AG preterm infants were compared to a group of low-risk pregnancies which we used to develop normal ranges of fetal renal parenchyma in a previous study.^20^

### Study process

Once recruited, infants were followed-up until discharge. A kidney ultrasound examination was performed at 32- and 37-weeks postmenstrual age (PMA). PMA was defined as:

PMA = Gestational age at birth in whole weeks + postnatal age in whole weeks.

The data of the fetal posterior renal parenchymal thickness at 32- and 37-weeks gestational age (GA) was compared to the extrauterine posterior renal parenchymal thickness of the preterm infants at the equivalent PMA obtained from this study. Infants with any renal abnormalities or other congenital or chromosomal abnormalities were excluded.

### Kidney ultrasound

The kidney ultrasound examinations were performed using a Philips Epiq or IU22 Ultrasound System (Philips Healthcare, Andover, MA) with a compact (small footprint) curved linear 5–8 MHz frequency transducer. All ultrasound examinations were performed by one of three Australian Accredited Medical Sonographers using a clearly defined protocol and who were blinded to the clinical information. B-mode imaging was utilized with the depth, gray-scale gain, focus, and time-gain compensation adjusted to acquire the best images of the kidneys. The image was magnified so the kidney occupied most of the image. The method used in this study is similar to our previous ultrasound study of the renal parenchyma, where the intra- and interobserver intra-class coefficient (ICC) was demonstrated to be greater than 95% for measurement of the renal parenchyma.^20^

**Fig. 1:**
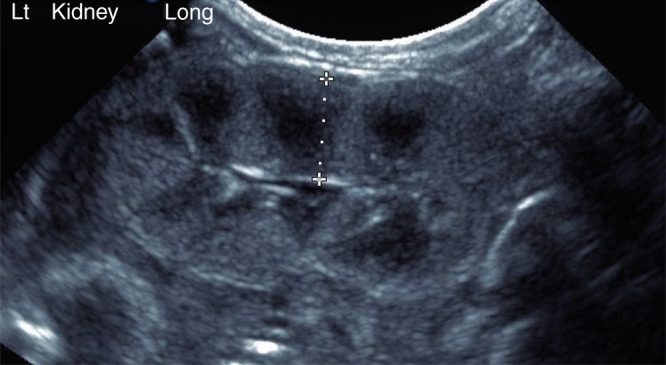
Neonatal kidney ultrasound. Sagittal ultrasound image of a preterm infant’s kidney at 32-weeks postmenstrual age with measurement of the renal parenchymal thickness of 7.2 mm.

Infants were examined in the prone position. A mid-sagittal view of the kidney was obtained, and the maximum length of the kidney was measured. The posterior parenchymal thickness was measured as the distance between the inner aspect of the posterior renal capsule and the sinus–pyramidal apex interface, in the middle third of the kidney (Fig. 1). The posterior parenchymal thickness was used as it was closest to the ultrasound transducer and our previous study demonstrated no significant difference between the anterior and posterior thickness.^20^ For each kidney, the measurement was performed twice and the mean of the two measurements was recorded. The fetal ultrasound measurements of the renal parenchyma were performed as outlined in our previous study of normal ranges of fetal renal parenchymal thickness.^20^ Pregnancy and birth outcomes, including GA at birth, birth weight, and sex were obtained from the electronic medical record.

### Statistical analysis

Sample size calculations were based on our previously published study demonstrating that the mean combined renal parenchymal thickness was 20 (±2) mm for term normal birth weight neonates and 18 (±3) mm for preterm infants at term correct age.^24^ Using a statistical power of 80% and a significance level of 0.05 (two-tailed) we calculated a total of at least 54 participants would be required (27 preterm infants and 27 fetuses).

R statistical language in R Studio (version 4.0.0) was used to analyze the data^25,26^ and the program ggplot2 was used to create the graph.^27^ The results are expressed as means ± standard deviation (SD) for continuous, normally distributed data and as medians (interquartile range (IQR)) for continuous, non-normally distributed data. To compare means of the renal parenchymal thickness between groups, independent *t*-tests (two-tailed) were used with *p* < 0.05 considered statistically significant.

## Results

**Fig. 2:**
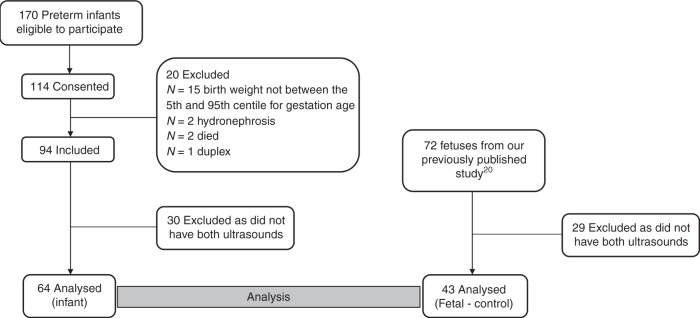
Participants. Flowchart of participant enrollment.

Of the 114 preterm infants recruited, two were excluded due to hydronephrosis, one was excluded with a duplex collecting system and two infants died. A further 15 infants were excluded as their birth weight was <5th centile or >95th centile for their GA (Fig. 2). Infants were also excluded (30) if they did not have both ultrasounds at 32- and 37-weeks PMA. Some infants did not have both ultrasounds if they were considered too unwell to have the investigation at the time the ultrasound was due, some were transferred back to regional hospitals and some went home and did not return for their 37-week PMA ultrasound. The data from 64 preterm infants were analyzed. Similarly, the participants in the fetal study^20^ had ultrasound scans approximately every four weeks and therefore not all participants had an ultrasound at the times analyzed for this study or they had delivered prior to 37 weeks. Therefore, after excluding 29 participants who did not have both ultrasounds, the data from 43 fetal ultrasounds were used as a control group to compare to the preterm infants. The birth characteristics of the infants are summarized in Table 1.

**Table 1:** Birth characteristics of the study population.

	Preterm (*N* = 64)	Fetal (*N* = 43)
GA at birth (weeks)	26.7 (24.7–28.6)	38.6 (38.2–39.3)
Birth weight (g)	935 (765–1312)	3360 (2912–3545)
Males	35 (54.7%)	28 (65.1%)

Data are given as median (interquartile range) or *N* (%).

*GA* gestational age.

**Fig. 3:**
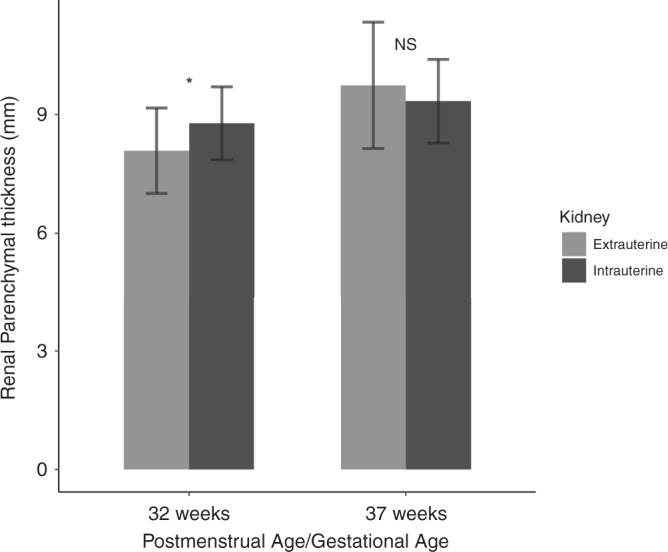
Bar graph of renal parenchymal thickness. Preterm infants (extrauterine) compared to fetuses (intrauterine) at 32- and 37-weeks postmenstrual age/gestational age. *p < 0.05; NS not significant.

Measurements of the posterior renal parenchymal thickness were obtained from both kidneys and then the average of the right and left kidneys were calculated. Table 2 summarizes the findings and Fig. 3 demonstrates the bar graph of the two groups at each age. The median GA of birth of the preterm infants was 26 weeks (IQR = 24–28). Therefore, at 32-weeks PMA, after around 6 weeks extrauterine kidney growth, the mean renal parenchymal thickness of the preterm infants was significantly thinner than the renal parenchymal thickness of the undelivered fetuses at 32-weeks GA (*t* (105) = −3.426, *p* = 0.0008). However, at 37-weeks PMA, after approximately eleven weeks extrauterine growth, there was no significant difference in the renal parenchymal thickness between the two groups. Between 32 and 37 weeks, the thickness of the renal parenchyma in the preterm infants increased by a mean of 1.7 mm (20.9%) whereas, in the fetal group it increased by a mean of 0.6 mm (6.8%). At 32 weeks, there was no significant difference between the renal parenchyma of the right and left kidneys (*p* = 0.92), however, at 37 weeks, the renal parenchyma of the left kidney was significantly thicker than the right (*p* = 0.03). There was no significant difference in the thickness of the renal parenchyma between males and females in the preterm or fetal groups at 32-weeks (*p* = 0.86 and 0.94, respectively) or 37-weeks (*p* = 0.41 and 0.60 respectively).

**Table 2:** Comparison of the mean renal parenchymal thickness of extrauterine measurements of preterm infants and intrauterine measurements of fetuses at 32- and 37-weeks GA.

GA	Mean RPT preterm (mm) *N* = 64	Mean RPT fetal (mm) *N* = 43	df	*t-*value	*p*-value
32w	8.09 (1.08)	8.78 (0.92)	105	−3.426	0.0008*
37w	9.74 (1.60)	9.34 (1.06)	105	1.428	0.1562

Data are given as mean (SD).

*GA* gestational age, *w* weeks, *RPT* renal parenchymal thickness.

*p < 0.05.

## Discussion

This study used a non-invasive imaging technique to offer insights into extrauterine nephrogenesis and our data demonstrated that at 32-weeks PMA, after an approximately six weeks extrauterine growth, the preterm infants had a significantly thinner renal parenchyma compared to the intrauterine fetal measurements. This thinner renal parenchyma in the preterm infants implies they have fewer layers of nephrons and a reduced nephron endowment. In preterm infants nephrogenesis has been demonstrated to continue extrauterine for up to 40 days.^7^ It is, however, thought to be impacted by both prenatal and postnatal issues such as intrauterine and extrauterine growth restriction, nephrotoxic medications, hyperoxia, sepsis, and hemodynamic factors in the neonatal care of the infant.^7,9,28^ Our findings are supported by autopsy studies demonstrating preterm infants had a thinner nephrogenic zone with fewer layers of glomeruli.^7,29^ Studies using ultrasound to assess renal volume also suggest that preterm infants have fewer nephrons.^5,15^

However, by 37-weeks PMA, the renal parenchymal thickness of the preterm infants was no longer significantly thinner than the fetal renal parenchyma at the equivalent GA. This suggests some extrauterine acceleration of the growth of the renal parenchyma. We postulate that this normalization of the renal parenchymal thickness seen in the preterm infants between 32- and 37-weeks PMA is possibly due to changes in the nephrogenic zone caused by compensatory hyperfiltration which is necessary to account for a deficit in nephron numbers. It is thought that over time, to maintain an adequate glomerular filtration rate and renal function, the existing nephrons undergo compensatory glomerular hyperfiltration.^9,30^ This hyperfiltration is linked with glomerular and tubular hypertrophy.^28,31^

Persistent glomerular hyperfiltration increases intra-glomerular capillary pressures which in turn causes the glomerular basement membrane to increase and glomerulomegaly to occur.^11,32^ Additionally, mechanical pressure on the post-filtration structures, causes dilation of the glomerular and tubular urinary spaces.^32,33^ Pre-clinical and autopsy studies demonstrate that prematurity is associated with significant numbers of abnormal glomeruli, with an enlarged Bowman’s space and a shrunken glomerular tuft.^7,11,12^ An autopsy study of over 60 infants, found there was an acceleration of renal maturation with glomerulomegaly and up to 13% of glomeruli in the kidney being abnormal in the preterm infants.^11^ Cullen-McEwen et al. demonstrated in a mouse model, that a reduction in glomerular number results in compensatory glomerular hyperfiltration and glomerulomegaly to maintain GFR.^34^ Evidence of higher single nephron glomerular filtration rate has been shown in studies of low birth weight and preterm infants.^14,35^ Additionally, in a previous study we found that despite the smaller kidney volumes of the preterm infant group at term corrected age when compared to the term control group, the renal parenchyma to renal volume ratio was greater in the preterm group which may have been, albeit partially, due to glomerulomegaly.^24^

This apparent “catch-up” and extrauterine increase of the thickness of the renal parenchyma of preterm infants between 32- and 37-weeks PMA may therefore be due to a combination of glomerulomegaly and hypertrophy of the proximal tubules, along with other interstitial structures within the parenchyma which are not yet clearly known. It is important to note that our study is a clinical study and therefore it is unable to directly demonstrate any components of impaired nephrogenesis. Rather, it provides non-invasive, clinical imaging which supports the pre-clinical and autopsy studies that demonstrate preterm birth does impair nephrogenesis.

Starting life with a reduced nephron number leads to an increased demand on the remaining nephrons. With further kidney insults during life, there can be further impacts on renal function and an increasing likelihood of developing CKD and hypertension. There is a need to optimize extrauterine renal development by seeking to modify factors that impact nephrogenesis in the extrauterine environment. Intervention would support the development of maximum nephron numbers and quality early in life to sustain optimal renal function throughout life. The impact of prematurity on extrauterine nephrogenesis and its potential impact on the long-term health of the infant should create awareness among clinicians to be more judicious on using potential nephrotoxic treatment options, such as aminoglycosides and non-steroidal anti-inflammatory drugs, while the infants are in the neonatal intensive care unit.^36–38^ Tools such as measuring the renal parenchymal thickness may assist in identifying treatments that may have the least detrimental effect on renal growth and function.

Our study did not assess the different factors that may have precipitated the preterm birth or the many conditions and factors of the postnatal environment which can potentially disrupt kidney development and result in a decrease in the quantity and quality of nephrons. The focus of the study was to investigate the use of ultrasound to non-invasively measure the renal parenchymal thickness to evaluate extrauterine kidney growth. The study had a relatively small cohort size with some loss of follow-up due to transfers back to regional hospitals.

## Conclusion

To our knowledge, this is the first study to compare extrauterine renal parenchymal thickness to intrauterine thickness of fetuses at the same gestation. The purpose of our study was to evaluate the extrauterine growth of the renal parenchyma of AG preterm infants and compare this to intrauterine renal parenchymal growth of a control group of low-risk pregnancies. At 32-weeks PMA the delivered preterm infants had a thinner parenchyma suggesting a reduced nephron endowment, however, by 37-weeks PMA there was an apparent “catch-up” of parenchymal growth in the preterm infants. It is plausible that a reduced nephron number results in compensatory hyperfiltration which likely causes hypertrophy of the glomeruli and tubules and an increase in interstitial mass and parenchymal thickness. More research is required to improve our understanding of how preterm birth affects nephrogenesis and kidney development overall and how non-invasive in-vivo tools, such as renal parenchymal thickness can aid in identifying factors that may be detrimental to nephrogenesis.
